# Oxaliplatin‐Induced Hypersensitivity Reactions Resulting in Respiratory and Cardiac Arrest: A Rare Case

**DOI:** 10.1002/ccr3.70989

**Published:** 2025-10-20

**Authors:** Qiaoyun Zuo, Eddile Zhang, Yingmei Li, Hua Zhang

**Affiliations:** ^1^ Department of Pharmacy The Seventh Affiliated Hospital, Sun Yat‐sen University Shenzhen Guangdong China; ^2^ Digestive Medicine Center The Seventh Affiliated Hospital, Sun Yat‐sen University Shenzhen Guangdong China

**Keywords:** case report, hypersensitivity reactions, oxaliplatin, respiratory and cardiac arrest

## Abstract

Oxaliplatin‐induced hypersensitivity reactions (HSRs), though rare, can cause life‐threatening cardiopulmonary arrest. This case underscores the importance of early identification of at‐risk patients by oncologists, nurses, and pharmacists. Timely intervention and vigilance, particularly through effective multidisciplinary collaboration, facilitate the recognition and management of acute cardiac toxicity, prevent severe cardiovascular events, reduce chemotherapy‐related allergic complications, and improve patient outcomes.

## 1 Introduction

Oxaliplatin is a third‐generation platinum‐based chemotherapy agent widely used in the treatment of colorectal and other gastrointestinal cancers [1]. While generally well tolerated, oxaliplatin is associated with adverse reactions including gastrointestinal, hematologic, and peripheral neurotoxic effects, though hypersensitivity reactions (HSRs) are increasingly recognized in 10%–25% of patients, particularly beyond six treatment cycles [2, 3, 4, 5]. These reactions are typically mild to moderate, presenting with symptoms such as flushing, rash, or shortness of breath [2, 6]. However, in rare cases, oxaliplatin‐induced HSRs can escalate rapidly and result in severe, life‐threatening complications, including respiratory and cardiac arrest [3]. Early identification and management are critical but often challenging due to the unpredictable nature of these reactions. We present a rare case of oxaliplatin‐induced HSR resulting in cardiopulmonary arrest shortly after infusion, aiming to highlight the clinical features, management approach, and considerations for early recognition and prevention of such events.

## 2 Case History/Examination

The patient was a 67‐year‐old male with no prior history of food or drug allergies. He was admitted to our facility for adjuvant chemotherapy following a diagnosis of rectal cancer. Over 3 years prior, he had undergone radical resection of the anorectal canal accompanied by a colostomy and had completed eight cycles of capecitabine combined with oxaliplatin without experiencing any HSRs.

## 3 Differential Diagnosis, Investigations, and Treatment

Before beginning oxaliplatin chemotherapy, the patient received several medications. These included a single intravenous dose of dexamethasone sodium phosphate (5 mg in 10 mL of 5% glucose), 40 mg of diphenhydramine administered intramuscularly, 40 mg of omeprazole given intravenously in 100 mL of 0.9% sodium chloride, and 5 mg of tropisetron hydrochloride administered intravenously in 100 mL of 0.9% sodium chloride.

The patient underwent relevant examinations from day 1 to day 4 after admission and made the necessary preparations for chemotherapy. On the 5th day of hospitalization at 17:46, the patient received oxaliplatin (Aiheng) at a dose of 130 mg/m^2^ (total dose: 220 mg, calculated based on body surface area of 1.72 m^2^ derived from patient weight 61 kg and height 173 cm using the Du Bois formula). The drug was diluted in 500 mL of 5% glucose and administered as a continuous intravenous infusion over 4 h (infusion rate: 125 mL/h), in strict accordance with the manufacturer's recommended infusion duration of 2–6 h for oxaliplatin. Two minutes after the infusion began, the patient lost consciousness and became unresponsive, so the medication infusion was stopped immediately. The examination revealed cyanosis lip and cold, clammy extremities. There was no rash on the body. Both pupils were dilated to approximately 6 mm and had a sluggish reaction to light. Auscultation revealed weak respiratory sounds in both lungs, and the heart monitor showed a heart rate of 42 beats per minute, blood pressure of 31 mmHg (systolic)/14 mmHg (diastolic), and blood oxygen saturation of 87%. The physician urgently consulted cardiology, anesthesiology, and intensive care unit (ICU), and immediately initiated cardiopulmonary resuscitation (CPR) and bedside suctioning, followed by the administration of 0.5 mg intramuscular epinephrine (once), 1 mg intravenous epinephrine (twice), and 12 mg intravenous norepinephrine. They administered 500 mL of succinylated gelatin and 500 mL of sodium lactate Ringer's solution via intravenous (IV) infusion for fluid resuscitation, along with 40 g of human albumin. At 18:12, the anesthesiologist inserted an endotracheal tube and assisted the patient's breathing with a bag‐valve mask. At 18:25, arterial blood gas analysis revealed a pH of 7.05 and a potassium level of 2.9 mmol/L. Based on the results in Table 1, the patient presented with both metabolic acidosis and respiratory acidosis. This prompted the administration of 250 mL of sodium bicarbonate intravenously to correct acidosis and 3 g of 15% potassium chloride to replenish potassium. At 18:42, the bedside electrocardiogram showed that he had atrial fibrillation with a rapid ventricular rate, with a heart rate of 168–200 beats per minute. The cardiologist performed synchronized electrical cardioversion at 150 J and 200 J at the bedside, which brought back a normal heart rhythm and a heart rate decrease to 110–120 beats per minute, with the addition of 0.6 g of esmolol intravenously to control the heart rate. At 19:06, a repeat arterial blood gas analysis showed a pH of 7.36 and potassium level of 2.9 mmol/L. At 20:04, his blood pressure improved to 124/72 mmHg. Consequently, norepinephrine support was withdrawn. Subsequent point‐of‐care ultrasonography revealed a severely reduced left ventricular ejection fraction (LVEF) of 31%, while the N‐terminal pro‐B‐type natriuretic peptide (NT‐proBNP) level was measured at 61.7 pg/mL (within normal reference range, < 125 pg/mL), as documented in Table 2. The examination revealed that the pupils measured approximately 3 mm and exhibited normal light reflexes. At 20:11, the patient regained self‐sustained breathing, indicating successful resuscitation.

**TABLE 1:** The results of arterial blood gas analysis.

Time	Temp (°C)	FiO_2_ (%)	PH	PaCO_2_ (mmHg)	PaO_2_(mmHg)	SaO_2_ (%)	lacticacid (mmol/L)	AB (mmol/L)	SB (mmol/L)	BE (mmol/L)	Kalium (mmol/L)
	36.3–37.2	—	7.35–7.45	35–48	83–108	95–98	< 1.3	21–28	21–28	−3.0 to +3.0	3.4–4.5
Day 5 18:25	35.8	61.0	7.05	48	177	98.9	11.3	13.8	11.8	−16.9	2.9
Day 5 19:06	36.3	61.0	7.36	46	388	99.3	10.6	25.9	24.9	−0.1	2.9
Day 5 20:10	37.0	61.0	7.09	92	255	99.5	7.8	27.9	21.8	−4.0	3.3

Abbreviations: AB, actual bicarbonate; BE, base excess; Dec, December; FiO_2_, fractional inspired oxygen; PaCO_2_, partial pressure of carbon dioxide; PaO_2_, partial pressure of oxygen; SaO_2_, oxygen saturation; SB, standard bicarbonate; Temp, temperature.

**TABLE 2:** Vital sign and laboratory data.

Variable	Reference range	Day 1	Day 5
Blood pressure (mmHg)	90–139/60–89	115/75	113/73 (Before administration)
Heart rate (Times/min)	60–100	85	82
Temperature (°C)	36–37	36.4	36.8
Respiration rate (Times/min)	12–28	18	19
Hematocrit (%)	40.00–50.00	31.10	31.40
Hemoglobin (g/L)	130.00–175.00	104.00	104.00
White‐cell count (×10^9^/L)	3.50–9.50	2.71	11.80
Differential count (×10^9^/L)			
Neutrophils	1.80–6.30	1.74	9.99
Lymphocytes	1.10–3.20	0.74	1.34
Monocytes	0.10–0.60	0.20	0.42
Eosinophils	0.02–0.52	0.02	0.04
Basophils	0.00–0.06	0.01	0.01
Platelet count (×10^9^/L)	125.00–350.00	75.00	83.00
Creatinine (μmol/L)	57.00–111.00	85.80	93.90
Alamine aminotransferase (U/L)	9.00–60.00	17.00	25.78
Aspartate aminotransferase (U/L)	15.00–45.00	9.00	27.32
Total protein (g/L)	65.00–85.00	73.92	52.41
Albumin (g/L)	40.00–55.00	38.50	24.9
Kalium (mmol/L)	3.50–5.30	3.89	3.41
Sodium (mmol/L)	137.00–147.00	140.00	140.00
Calcium (mmol/L)	2.20–2.50	2.29	2.12
N‐terminal pro B‐type natriuretic peptide (NT‐proBNP) (pg/mL)	< 125	/	61.7

## 4 Conclusion and Results (Outcome and Follow‐Up)

In spite of his critical state, the patient's family strongly requested discharge. However, the attending physician informed them that the patient's condition was critical and advised continued hospitalization for treatment. The physician warned that during the discharge or transfer process, there was a possibility of unexpected events occurring, which might prevent the patient from receiving timely rescue and treatment. This could potentially lead to serious adverse outcomes and pose a threat to the patient's life. Despite these warnings, the patient's family voluntarily assumed the risks and consequences associated with discharging against medical advice. They signed an automatic discharge consent form before the discharge was processed. Consequently, he was gradually weaned off tracheal ventilation. Arterial blood gas analysis results, as detailed in Table 1, indicated a significant improvement in metabolic acidosis; however, due to diminished respiratory function following resuscitation, the patient was discharged with respiratory acidosis. The blood levels of electrolytes were almost normal, as were the results of tests of kidney function and liver. Vital sign and other laboratory test results are shown in Table 2.

## 5 Discussion

### 5.1 The Incidence and Risk Factors of Hypersensitivity Reactions Induced by Oxaliplatin

HSRs to oxaliplatin have emerged as a significant clinical concern [4], particularly in the context of its increasing use in the treatment of colorectal cancer and other malignancies [5]. Recent studies indicate that oxaliplatin‐induced hypersensitivity reactions occur in 11.7%–19% of patients, with severe (grade 3/4) reactions observed in 1.6%–2.2% of cases. Notably, most reactions are mild to moderate (grade 1–2) [7, 8]. A history of allergies and the interval without oxaliplatin are independent risk factors for HSRs to oxaliplatin in colorectal cancer patients [9]. Being male and having higher peripheral eosinophil counts are risk factors for oxaliplatin‐induced HSRs, which can be managed through desensitization protocols [10].

### 5.2 Possible Pathological Mechanisms and Clinical Manifestations of Hypersensitivity Reactions to Oxaliplatin

The HSRs induced by oxaliplatin involve multi‐layered pathological mechanisms. In terms of genetic susceptibility, studies have shown that the HLA‐DRB1*12:01 allele significantly increases the risk of oxaliplatin‐induced immune‐mediated HSRs (OR = 3.4–12.0), suggesting a key role of HLA class II molecules in antigen presentation [11]. Regarding immune cell activation, basophil activation tests (BAT) indicate that the activity of CD63^+^ and CD203c^+^ basophils is significantly elevated in HSR patients, with CD203c showing higher sensitivity to oxaliplatin stimulation, supporting the involvement of a non‐IgE‐dependent mechanism [11, 12]. Although specific IgE to oxaliplatin can be detected in a small number of patients (8.5%) [11], the low positive rate suggests that mast cells may be directly activated through pathways such as Mas‐related G protein‐coupled receptor X2 (MRGPRX2) [11]. Additionally, T cell‐mediated delayed‐type reactions may be related to clonal expansion triggered by drug‐HLA complexes and reactivation of herpes viruses, leading to systemic inflammatory responses [13, 14]. Overall, the pathological mechanisms of oxaliplatin HSRs encompass genetic risk, innate immune activation, and adaptive immune responses.

Oxaliplatin‐induced hypersensitivity reactions (HSRs) typically exhibit a spectrum of clinical manifestations, ranging from mild cutaneous eruptions to life‐threatening anaphylaxis [2, 3, 6]. However, this rare case illustrates the unpredictable nature of oxaliplatin‐induced hypersensitivity reactions (HSRs), presenting as immediate cardiopulmonary collapse without classic allergic prodromes (rash, eosinophilia, or respiratory distress) in a 67‐year‐old male with no prior allergy history. Despite completing 8 uneventful cycles of XELOX therapy, the patient developed sudden loss of consciousness and cardiorespiratory arrest within 2 min of the ninth oxaliplatin infusion, aligning with ICD‐9 criteria for unspecified adverse drug effects (995.2) rather than typical anaphylaxis (995.0) or allergic presentations (995.3) [15, 16]. Such rapid‐onset, non‐IgE‐mediated toxicity challenges conventional risk stratification models that prioritize allergy history (OR = 2.232) and classical symptoms (75.4% pruritus, 60% dyspnea prevalence) [17]. This patient developed a life‐threatening hypersensitivity reaction, which was successfully reversed with prompt resuscitation, restoring spontaneous respiration. However, following the family's decision to pursue discharge against medical advice, the patient manifested respiratory acidosis—an underreported complication in oxaliplatin‐related hypersensitivity literature.

### 5.3 Hypersensitivity Reactions to Oxaliplatin: Prevention, Alternative Treatment Options and Management Strategies

Premedication with dexamethasone seems to be a protective factor for the development of mild reactions (Grade 1–2), together with the preventive administration of antihistamines before the infusion in order to minimize the risk of triggering an allergic reaction [6, 18]. To prevent allergic reactions, the patient is pre‐treated with 5 mg of dexamethasone and 40 mg of diphenhydramine prior to the administration of oxaliplatin. We believed that the preemptive administration of corticosteroids and antihistamines helped prevent visible symptoms (e.g., rashes) during hypersensitivity reactions. However, their use might also have made hypersensitivity reactions more challenging to identify due to the absence of typical cutaneous manifestations. Further large‐scale clinical studies were required to validate this hypothesis. The patient did not develop a rash after using oxaliplatin, but an anaphylactic reaction led to respiratory and cardiac arrest. Therefore, during the cardiopulmonary resuscitation process, the focus was on saving the patient's life, and corticosteroids and antihistamines were not used.

According to the NCCN guidelines for the diagnosis and treatment of rectal cancer, for patients who are not suitable for oxaliplatin treatment, capecitabine monotherapy or the 5‐fluorouracil/leucovorin (5‐FU/LV) regimen is recommended as an alternative treatment option [1]. Immune checkpoint inhibitors (pembrolizumab) provide a breakthrough alternative for patients with MSI‐H/dMMR metastatic colorectal cancer. The KEYNOTE‐177 trial showed that pembrolizumab significantly extended progression‐free survival (PFS) compared to chemotherapy ± targeted therapy (16.5 vs. 8.2 months), and had a higher objective response rate (ORR) (43.8% vs. 33.1%) [19]. For RAS/BRAF wild‐type patients, the combination of cetuximab with irinotecan or monotherapy with anti‐EGFR monoclonal antibodies may be beneficial [20].

The rising utilization of oxaliplatin in colorectal cancer necessitates heightened vigilance among oncology teams (oncologists, nurses, and pharmacists) regarding hypersensitivity reactions (HSRs) [21]. Current management protocols emphasize: institutional standardization of graded infusion adjustments, corticosteroid/antihistamine premedication [22, 23], and desensitization protocols; risk stratification via emerging biomarkers (HLA haplotypes/basophil activation testing) [11]; and structured patient education to address the 28.6% HSR recurrence risk upon rechallenge [8]. This case highlights critical action points: immediate infusion cessation combined with epinephrine/norepinephrine administration to prevent hemodynamic collapse, alongside protocol‐driven post‐resuscitation monitoring—particularly crucial when managing non‐adherent discharges. Mandatory staff training in anaphylaxis management and vital sign surveillance systems are strongly advocated to mitigate morbidity [24, 25, 26].

## 6 Conclusion

This case highlights the possibility of severe hypersensitivity reactions after oxaliplatin infusion, which necessitates particular vigilance from healthcare providers.

## Author Contributions

**Qiaoyun Zuo:** data curation, formal analysis, investigation, methodology, project administration, validation, writing – original draft, writing – review and editing. **Eddile Zhang:** supervision, validation, writing – review and editing. **Yingmei Li:** validation, writing – review and editing. **Hua Zhang:** methodology, methodology, project administration, project administration, supervision, supervision, validation, validation, writing – review and editing, writing – review and editing.

## Ethics Statement

The studies involving human participants were reviewed and approved by the Ethical Committee of the Seventh Affiliated Hospital, Sun Yat‐sen University. The patients/participants provided their written informed consent to participate in this study. Written informed consent was obtained from the individual(s) for the publication of any potentially identifiable images or data included in this article.

## Consent

Written informed consent was obtained from the patient to publish this report in accordance with the journal's patient consent policy.

## Conflicts of Interest

The authors declare no conflicts of interest.

## Data Availability

The data used to support the finding of this study are included within the article.
