# Correction to “Oxaliplatin‐Induced Hypersensitivity Reactions Resulting in Respiratory and Cardiac Arrest: A Rare Case”

**DOI:** 10.1002/ccr3.71423

**Published:** 2025-11-12

Zuo Q, Zhang E, Li Y, Zhang H “Oxaliplatin‐Induced Hypersensitivity Reactions Resulting in Respiratory and Cardiac Arrest: A Rare Case,” *Clinical Case Reports* (2025) 13 no. 10: e70989, https://doi.org/10.1002/ccr3.70989, 41127694.

In the originally published version of this article, the “Funding Information” section was inadvertently omitted during the submission process. The article currently states “**Funding:** The authors received no specific funding for this work.”

This should be updated to include the correct funding acknowledgment as follows: “**Funding:** This study was supported by the Seventh Affiliated Hospital, Sun Yat‐sen University Clinical Research 735 Program (Grant No. ZSQY202273513).

We apologize for this error.

